# Implications of end‐of‐life home care compared with hospital‐based care during the COVID‐19 pandemic: A case report

**DOI:** 10.1002/ccr3.5806

**Published:** 2022-07-18

**Authors:** Ryo Sakamoto, Divya Bhandari, Makoto Yoshida, Hirotomo Miyatake, Makoto Kosaka, Akihiko Ozaki, Tetsuya Tanimoto, Masahiro Kami

**Affiliations:** ^1^ Visina Home Care Tokyo Japan; ^2^ 536425 Medical Governance Research Institute Tokyo Japan; ^3^ Teikyo University School of Medicine Tokyo Japan; ^4^ Orange Home‐Care Clinic Fukui Japan; ^5^ Department of Breast Surgery Jyoban Hospital of Tokiwa Foundation Fukushima Japan

**Keywords:** homecare < social care, infectious diseases, nursing, psychiatry

## Abstract

Restriction on hospital visits for COVID‐19 infection control continues to have a significant negative impact on patients and their families. For a patient receiving palliative care, this social isolation may deteriorate their mental health. In such situations, home care could be a viable solution to this problem.

## Abstract 2

In the COVID‐19 pandemic, providing end‐of‐life care at home may allow patients to spend more time with their families, improve communication between them and their health professionals and reduce the possibility of hospital‐acquired infections.

## 1 INTRODUCTION

End‐of‐life care for cancer patients aimed to optimize the quality of life by alleviating physical, social, spiritual, and psychological suffering experienced throughout the dying process.^1^ Meaningful interaction and communication between terminally ill patients and their family members is crucial because it enables a patient to adjust to the dying process with ease and alleviates the distress of family members allowing them to move on after death.^1^

The current coronavirus disease 2019 (COVID‐19) pandemic has affected various aspects of healthcare, and end‐of‐life care is no exception. In an attempt to limit the spread of COVID‐19 infection, hospitals and nursing homes have imposed visiting restrictions, which prevent patients from spending precious time with their loved ones in their final days of life. This separation might further worsen the mental health of both vulnerable patients and their family members.^2^ Moreover, the pandemic has added another layer to the complexity of providing effective end‐of‐life care to cancer patients in hospitals, who are highly susceptible to the infection themselves.

Home care could be a preferable solution to this problem. Home care is a form of care in which patients stay at home with the help of their families, nurses, care workers, doctors and other health care professionals. This form of care is actively practiced in some countries, including Japan, where reimbursement for home care is available, including for patients with cancer and palliative care needs.^3^, ^4^ In Japan, a state of emergency due to COVID‐19 was declared in April 2020, and to date, the infection has not been well controlled. Hospitals have implemented restrictions on the number of people who can visit hospitalized patients. Therefore, home care can be a viable alternative option to deal with issues related to end‐of‐life care during the COVID‐19 pandemic. In this article, we are presenting a case study of a Japanese patient who spent the final stages of his illness receiving professional care at home during the COVID‐19 pandemic.

## 2 CASE PRESENTATION

A man in his late 80s living in Tokyo with his wife and two daughters experienced sudden abdominal pain in July 2020 and went to seek help in a nearby tertiary hospital. That same day, he underwent an emergency laparotomy and appendicectomy after being diagnosed with generalized peritonitis associated with the perforation of an appendiceal tumor. Once the diagnostic tests were conducted, the patient was confirmed to have Stage IV appendiceal cancer. Postoperative contrast‐enhanced computed tomography (CT) scan did not show any abnormal findings such as local recurrence, lymphadenopathy, or distant metastasis associated with cancer. However, histopathological tests revealed scattered lymphatic invasion in the sub‐serous layer. Accordingly, the patient was offered palliative care instead of invasive procedures such as surgery.

Family members were informed about the severity of the disease highlighting the need for end‐of‐life care. However, due to COVID‐19, the hospital imposed certain restrictions, which limited the number of visitors it can allow per patient. Only one family member was allowed to visit an inpatient, and only with approval from the hospital or physician. Accordingly, the family started seeking updates on the patient's condition through phone calls with the medical staff. However, family members were unable to comprehend all information clearly, leading to misunderstandings and even conflicts between healthcare staff and family members. For example, there was an incident where the family members thought that the intravenous drip was administered through the peripheral intravenous catheter, but later they found it was administered through the peripherally inserted central venous catheter. So, family members were having difficulty adjusting to such incidents. They were particularly concerned when hospital staff asked them not to call too often and were told that medical staff would contact them directly if anything happened to the patient.

The patient's family was worried about home care initially because the hospital told them it would be difficult and expensive. However, considering the patient's desire to spend his final period at home with his family, along with the restriction on hospitals visits due to the pandemic, the patient and his family chose to receive professional home care services in November 2020. After his discharge, he started receiving home care where home care nurses visited twice a day and the home doctor visited once every 2 weeks. Moreover, two daughters assisted his wife as family caregivers, and professional caregivers provided clinical care through a publicly funded nursing care insurance scheme. The patient was able to spend ample time with their loved ones, which was not possible in the hospital. On weekends, his grandchildren and great‐grandchildren also visited him, and the whole family was able to spend quality time with the terminally ill patient. Moreover, communication between the healthcare professionals and the family was also enhanced. Nurses communicated on a daily basis, and the doctor in charge made house calls as needed. In April 2021, the patient passed away peacefully after experiencing *acute respiratory distress syndrome (ARDS)* secondary to heart failure. The patient received well‐organized, proficient home‐based health care, and spent the final days of his life with his family, friends, and other loved ones.

## 3 DISCUSSION

This case illustrates the importance of home‐based end‐of‐life care for cancer patients, particularly during crises such as the COVID‐19 pandemic, as it allows patients to spend quality time with their families along with optimal care if managed effectively. Typically, end‐of‐life care is provided around the globe in hospice or hospital, aiming to provide holistic, personalized care to patients and to support families and caregivers.^5^ These facilities, however, have implemented visiting restrictions to prevent the spread of COVID‐19, which might lead to other adverse health consequences such as social isolation and emotional loneliness among patients.^6^

In our case, the terminally ill patient was able to enjoy the comfort of his own home, cherish his lifetime memories, and spend quality time with his loved ones. Besides the psychosocial implications, we believe that there are several other reasons to choose home‐based end‐of‐life care during a COVID‐19 pandemic. First, this would prevent communication problems between healthcare professionals and family members. Restrictions imposed due to COVID‐19 have reportedly reduced the quality of communication between hospitalized patients and their family members, resulting in severe distress that may affect the quality of death and bereavement.^7^, ^8^ Second, home care would potentially reduce the burden of COVID‐19 on our healthcare system. End‐of‐life patients are a vulnerable population who are more likely to experience serious consequences if infected with COVID‐19.^9^ Besides the potential risk of nosocomial infection, work overload, logistic, and staff shortages are also an issue in hospitals during this pandemic.^9^ Given these reasons, home care could be considered as a viable option, at least until the pandemic subsides. Further research is needed to determine the impact of home‐based care on outcomes for patients and their families.

However, home care also has its drawbacks. Previous studies have reported challenges related to delivering urgent care when necessary^10^ and difficulties in securing staff.^11^ Further, it cannot be denied that there may be a risk of COVID‐19 infection due to frequent visits from health workers, families, and relatives. In any case, patients must be informed about the advantages and disadvantages of hospital/hospice and home care so that they can make informed choices.

## 4 CONCLUSION

The findings of this case study highlight that in crises situations such as COVID‐19, home care could be considered as a viable option for terminally ill cancer patients.

## AUTHOR CONTRIBUTIONS

Sakamoto R, Bhandari D, Ozaki A, Yoshida M, and Tanimoto T wrote the manuscript. All authors conceptualized and designed the study and contributed to making critical revisions for improving the intellectual content of the manuscript.

## CONFLICT OF INTEREST

AO reports personal fees from Medical Network Systems, MNES Inc. TT reports personal fees from Medical Network Systems, MNES Inc. and Bionics co. Itd. Other authors declare no competing interests.

### ETHICAL APPROVAL

Written informed consent was obtained from the patient's child.

### CONSENT

The patient is deceased and we obtained a consent form from the patient's child.

## Data Availability

Data sharing is not applicable to this article as no datasets were generated or analyzed during current study.
